# The genome sequence of the wood-carving leafcutter bee, *Megachile ligniseca *(Kirby, 1802)

**DOI:** 10.12688/wellcomeopenres.21002.1

**Published:** 2024-03-01

**Authors:** Liam M. Crowley, Steven Falk, University of Oxford and Wytham Woods Genome Acquisition Lab, Darwin Tree of Life Barcoding collective, Wellcome Sanger Institute Tree of Life Management, Samples and Laboratory team, Wellcome Sanger Institute Scientific Operations: Sequencing Operations, Wellcome Sanger Institute Tree of Life Core Informatics team, Tree of Life Core Informatics collective, Darwin Tree of Life Consortium

**Affiliations:** 1 University of Oxford, Oxford, England, UK; 2 Independent researcher, Kenilworth, England, UK

**Keywords:** Megachile ligniseca, wood-carving leafcutter bee, genome sequence, chromosomal, Hymenoptera

## Abstract

We present a genome assembly from an individual female *Megachile ligniseca* (the wood-carving leafcutter bee; Arthropoda; Insecta; Hymenoptera; Megachilidae). The genome sequence is 290.0 megabases in span. Most of the assembly is scaffolded into 16 chromosomal pseudomolecules. The mitochondrial genome has also been assembled and is 23.71 kilobases in length. Gene annotation of this assembly on Ensembl 11,722 protein coding genes.

## Species taxonomy

Eukaryota; Opisthokonta; Metazoa; Eumetazoa; Bilateria; Protostomia; Ecdysozoa; Panarthropoda; Arthropoda; Mandibulata; Pancrustacea; Hexapoda; Insecta; Dicondylia; Pterygota; Neoptera; Endopterygota; Hymenoptera; Apocrita; Aculeata; Apoidea; Anthophila; Megachilidae; Megachilinae; Megachilini; *Megachile*; *Megachile ligniseca* (Kirby, 1802) (NCBI:txid1542540).

## Background

**Figure 1.**
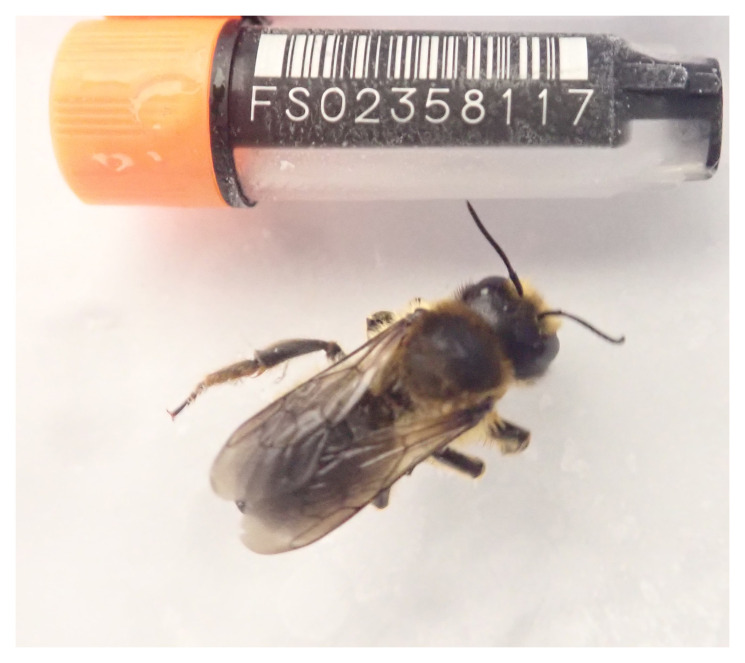
Photograph of the *Megachile ligniseca* (iyMegLign2) specimen used for Hi-C sequencing.

The Wood-carving Leafcutter Bee, *Megachile ligniseca* ( Figure 1), is a large (forewing length 8.5–12 mm), brown, solitary bee in the family Megachilidae. It occurs across northern and central Europe. In the UK is has a south-eastern distribution, extending north to Yorkshire and west into south Wales. Females have a buff-coloured scopal hairs with the hairs of the final two sternites black. Tergite six is rounded and lacks erect hairs. Males have a deep notch on the posterior margin of tergite six and the fore-tarsi are unmodified.

It can be found in a range of habitats, including woodlands, scrub and gardens, and particularly post-industrial and brownfield sites ( Falk & Lewington, 2019). It is a solitary, univoltine species with a flight period from June to September, peaking in July to August. Nests are constructed in aerial cavities, typically in holes in dead wood although artificial cavities and hollow plant stems are also used ( Heroldovê *et al.*, 2021). Females cut sections of leaves and occasionally petals that are used to wrap individual cells and to partition and seal nests. Adults visit a range of flowers including thistles, bramble and Himalayan balsam ( *Impatiens glandulifera*) ( Gresty *et al.*, 2018), and nectar rob from, occasionally destroying, deep flowers ( Wildermuth & Krebs, 2010).

The complete genome sequence for this species will facilitate studies into the evolution of sociality, reproductive systems and Hymenopteran taxonomy.

## Genome sequence report

The genome was sequenced from one female *Megachile ligniseca* collected from Wytham Woods, Oxfordshire (biological vice-county Berkshire), UK (51.77, –1.34). A total of 80-fold coverage in Pacific Biosciences single-molecule HiFi long reads was generated. Primary assembly contigs were scaffolded with chromosome conformation Hi-C data. Manual assembly curation corrected one missing join.

**Figure 2.**
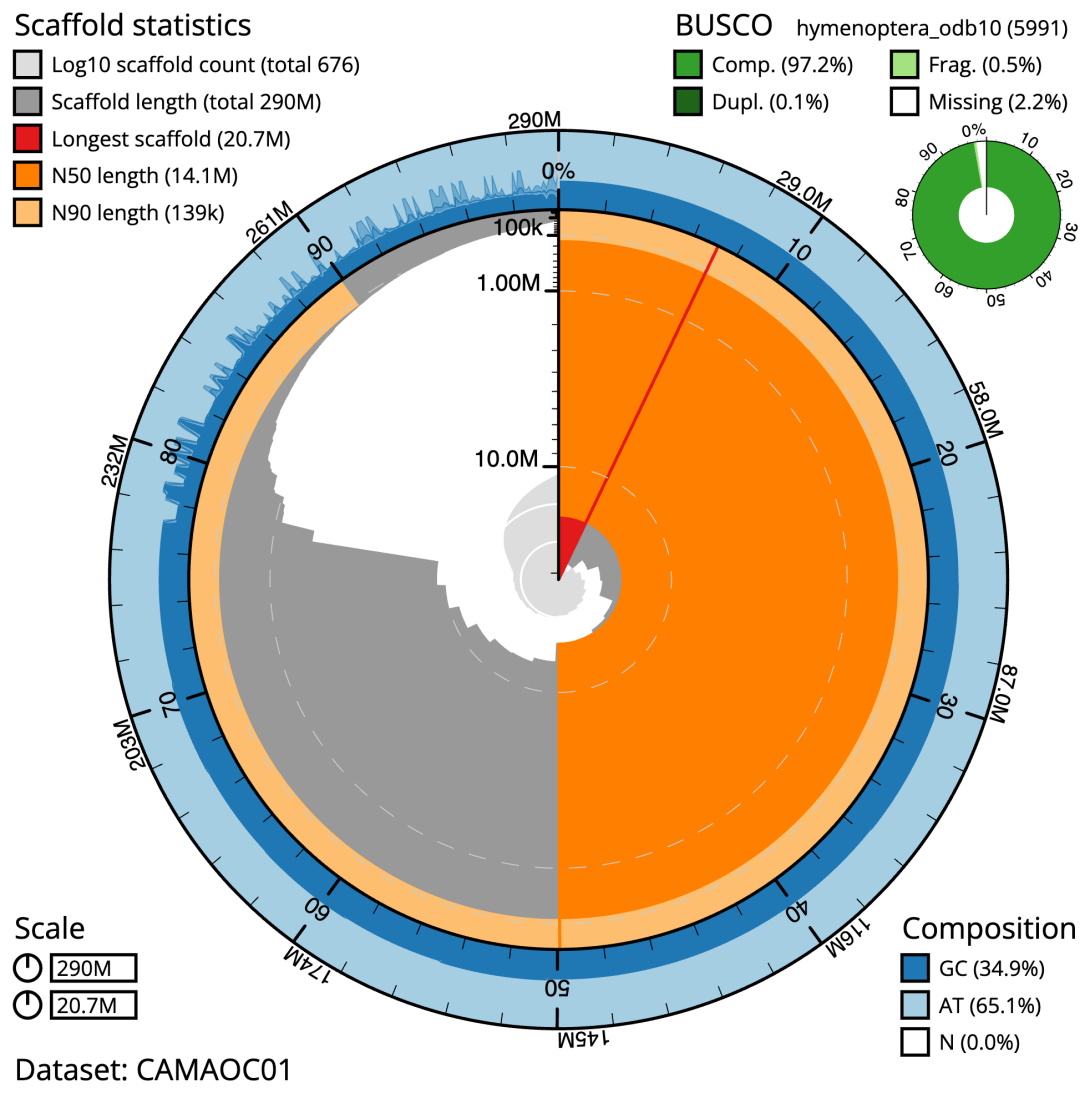
Genome assembly of *Megachile ligniseca*, iyMegLign1.1: metrics. The BlobToolKit Snailplot shows N50 metrics and BUSCO gene completeness. The main plot is divided into 1,000 size-ordered bins around the circumference with each bin representing 0.1% of the 290,044,579 bp assembly. The distribution of scaffold lengths is shown in dark grey with the plot radius scaled to the longest scaffold present in the assembly (20,718,490 bp, shown in red). Orange and pale-orange arcs show the N50 and N90 scaffold lengths (14,111,993 and 139,481 bp), respectively. The pale grey spiral shows the cumulative scaffold count on a log scale with white scale lines showing successive orders of magnitude. The blue and pale-blue area around the outside of the plot shows the distribution of GC, AT and N percentages in the same bins as the inner plot. A summary of complete, fragmented, duplicated and missing BUSCO genes in the hymenoptera_odb10 set is shown in the top right. An interactive version of this figure is available at https://blobtoolkit.genomehubs.org/view/CAMAOC01/dataset/CAMAOC01/snail.

**Figure 3.**
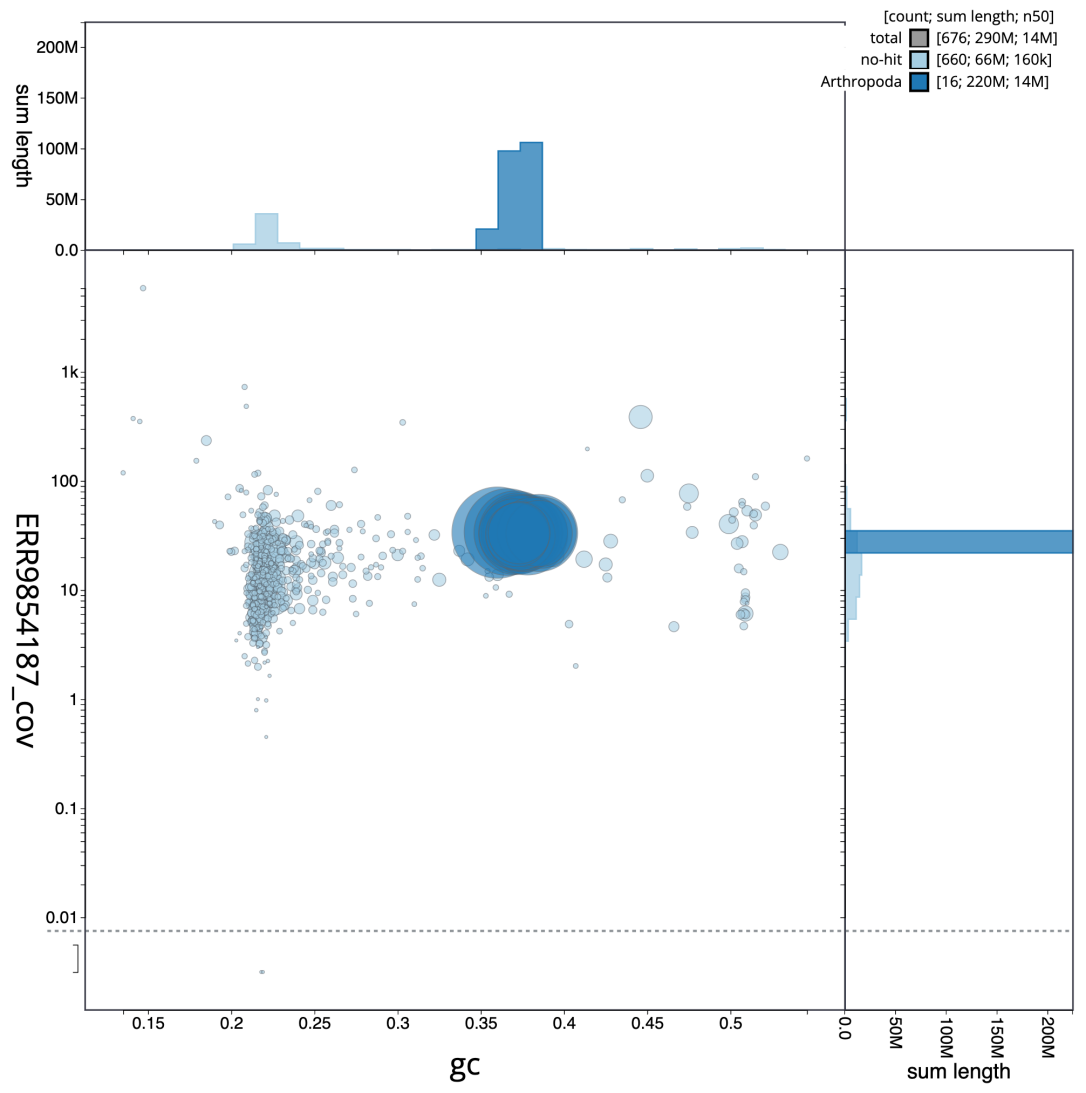
Genome assembly of *Megachile ligniseca*, iyMegLign1.1: BlobToolKit GC-coverage plot. Scaffolds are coloured by phylum. Circles are sized in proportion to scaffold length. Histograms show the distribution of scaffold length sum along each axis. An interactive version of this figure is available at https://blobtoolkit.genomehubs.org/view/CAMAOC01/dataset/CAMAOC01/blob.

**Figure 4.**
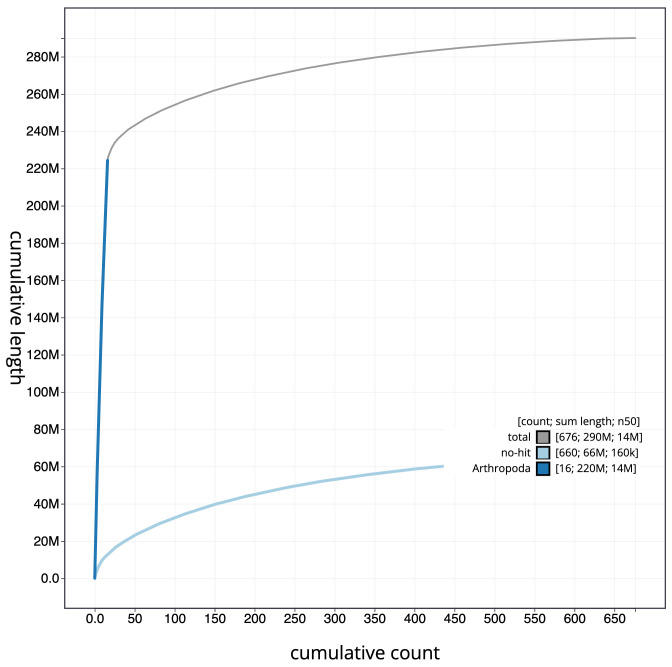
Genome assembly of *Megachile ligniseca*, iyMegLign1.1: BlobToolKit cumulative sequence plot. The grey line shows cumulative length for all scaffolds. Coloured lines show cumulative lengths of scaffolds assigned to each phylum using the buscogenes taxrule. An interactive version of this figure is available at https://blobtoolkit.genomehubs.org/view/CAMAOC01/dataset/CAMAOC01/cumulative.

**Figure 5.**
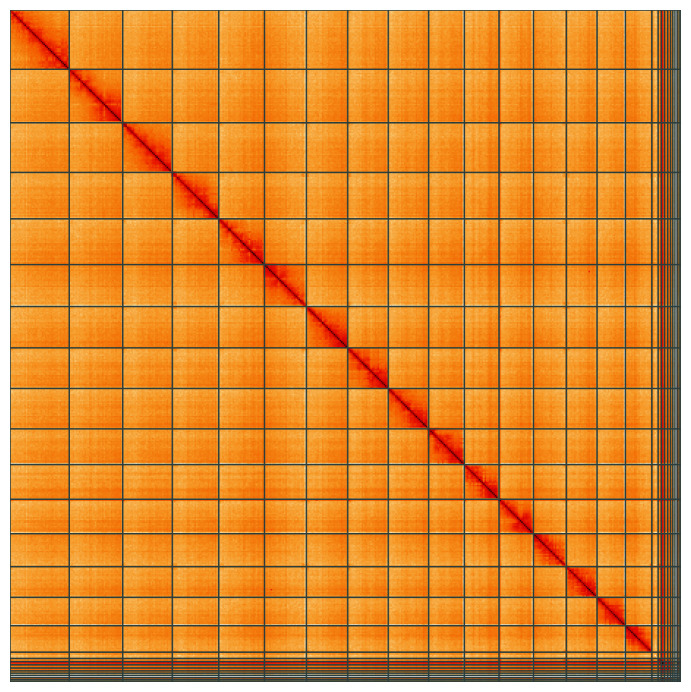
Genome assembly of *Megachile ligniseca*, iyMegLign1.1: Hi-C contact map of the iyMegLign1.1 assembly, visualised using HiGlass. Chromosomes are shown in order of size from left to right and top to bottom. An interactive version of this figure may be viewed at https://genome-note-higlass.tol.sanger.ac.uk/l/?d=LsvkNZzWSdmDtY48U4dLfA.

The final assembly has a total length of 290.0 Mb in 675 sequence scaffolds with a scaffold N50 of 14.1 Mb ( Table 1). The snailplot in Figure 2 provides a summary of the assembly statistics, while the distribution of assembly scaffolds on GC proportion and coverage is shown in Figure 3. The cumulative assembly plot in Figure 4 shows curves for subsets of scaffolds assigned to different phyla. Most (77.35%) of the assembly sequence was assigned to 16 chromosomal-level scaffolds. Chromosome-scale scaffolds confirmed by the Hi-C data are named in order of size ( Figure 5; Table 2). While not fully phased, the assembly deposited is of one haplotype. Contigs corresponding to the second haplotype have also been deposited. The mitochondrial genome was also assembled and can be found as a contig within the multifasta file of the genome submission.

**Table 1.** Genome data for *Megachile ligniseca*, iyMegLign1.1.

Project accession data		
Assembly identifier	iyMegLign1.1	
Species	*Megachile ligniseca*	
Specimen	iyMegLign1	
NCBI taxonomy ID	1542540	
BioProject	PRJEB53608	
BioSample ID	SAMEA7520495	
Isolate information	iyMegLign1, female: thorax (DNA sequencing) iyMegLign2, female: head and thorax (Hi-C sequencing) iyMegLign3, male: abdomen (RNA sequencing)	
Assembly metrics *		*Benchmark*
Consensus quality (QV)	59.2	*≥ 50*
*k*-mer completeness	100.0%	*≥ 95%*
BUSCO **	C:97.2%[S:97.1%,D:0.1%], F:0.5%,M:2.2%,n:5,991	*C ≥ 95%*
Percentage of assembly mapped to chromosomes	77.35%	*≥ 95%*
Sex chromosomes	None	*localised homologous pairs*
Organelles	Mitochondrial genome: 23.71 kb	*complete single alleles*
Raw data accessions		
PacificBiosciences SEQUEL II	ERR9854186, ERR9854187	
Hi-C Illumina	ERR9866451	
PolyA RNA-Seq Illumina	ERR10890684	
Genome assembly		
Assembly accession	GCA_945859555.1	
*Accession of alternate haplotype*	GCA_945859565.1	
Span (Mb)	290.0	
Number of contigs	701	
Contig N50 length (Mb)	14.1	
Number of scaffolds	675	
Scaffold N50 length (Mb)	14.1	
Longest scaffold (Mb)	20.72	
Genome annotation		
Number of protein-coding genes	11,722	
Number of non-coding genes	3,984	
Number of gene transcripts	31,862	

* Assembly metric benchmarks are adapted from column VGP-2020 of “Table 1: Proposed standards and metrics for defining genome assembly quality” from Rhie *et al.* (2021). ** BUSCO scores based on the hymenoptera_odb10 BUSCO set using version 5.3.2. C = complete [S = single copy, D = duplicated], F = fragmented, M = missing, n = number of orthologues in comparison. A full set of BUSCO scores is available at https://blobtoolkit.genomehubs.org/view/CAMAOC01/dataset/CAMAOC01/busco.

**Table 2.** Chromosomal pseudomolecules in the genome assembly of *Megachile ligniseca*, iyMegLign1.

INSDC accession	Chromosome	Length (Mb)	GC%
OX243777.1	1	20.72	36.0
OX243778.1	2	18.67	36.5
OX243779.1	3	17.34	37.0
OX243780.1	4	16.22	37.5
OX243781.1	5	16.01	38.0
OX243782.1	6	14.64	38.5
OX243783.1	7	14.44	37.5
OX243784.1	8	14.19	37.5
OX243785.1	9	14.11	37.5
OX243786.1	10	12.48	38.5
OX243787.1	11	12.11	37.0
OX243788.1	12	12.03	38.0
OX243789.1	13	11.52	37.5
OX243790.1	14	10.7	38.5
OX243791.1	15	9.91	37.0
OX243792.1	16	9.26	37.5
OX243793.1	MT	0.02	15.0

The estimated Quality Value (QV) of the final assembly is 59.2 with *k*-mer completeness of 100.0%, and the assembly has a BUSCO v5.3.2 completeness of 97.2% (single = 97.1%, duplicated = 0.1%), using the hymenoptera_odb10 reference set ( *n* = 5,991).

Metadata for specimens, barcode results, spectra estimates, sequencing runs, contaminants and pre-curation assembly statistics are given at https://links.tol.sanger.ac.uk/species/1542540.

## Genome annotation report

The *Megachile ligniseca* genome assembly (GCA_945859555.1) was annotated by Ensembl at the European Bioinformatics Institute (EBI) using the Ensembl rapid annotation pipeline ( Table 1; https://rapid.ensembl.org/Megachile_ligniseca_GCA_945859555.1/Info/Index). The resulting annotation includes 31,862 transcribed mRNAs from 11,722 protein-coding and 3,984 non-coding genes.

## Methods

### Sample acquisition and nucleic acid extraction

A female *Megachile ligniseca* (specimen ID Ox000134, ToLID iyMegLign1) was collected from Wytham Woods, Oxfordshire (biological vice-county Berkshire), UK (latitude 51.77, longitude –1.34) on 2019-08-07 by netting. The specimen used for Hi-C sequencing (specimen ID Ox000489, ToLID iyMegLign2) was netted in the same location on 2020-06-15. The specimens were collected and identified by Liam Crowley (University of Oxford). A third specimen (specimen ID Ox000744, ToLID iyMegLign3), used for RNA sequencing, was collected from Wytham Woods on 2020-08-03. This specimen was collected and identified by Steven Falk (independent researcher). All specimens were snap-frozen on dry ice before processing.

Protocols developed by the Wellcome Sanger Institute (WSI) Tree of Life core laboratory have been deposited on protocols.io ( Denton *et al.*, 2023b). The workflow for high molecular weight (HMW) DNA extraction at the WSI includes a sequence of core procedures: sample preparation; sample homogenisation, DNA extraction, fragmentation, and clean-up. In sample preparation, the iyMegLign1 sample was weighed and dissected on dry ice ( Jay *et al.*, 2023). Tissue from the head and thorax was homogenised using a PowerMasher II tissue disruptor ( Denton *et al.*, 2023a).

HMW DNA was extracted using the Automated MagAttract v1 protocol ( Sheerin *et al.*, 2023). HMW DNA was sheared into an average fragment size of 12–20 kb in a Megaruptor 3 system with speed setting 30 ( Todorovic *et al.*, 2023). Sheared DNA was purified by solid-phase reversible immobilisation ( Strickland *et al.*, 2023): in brief, the method employs a 1.8X ratio of AMPure PB beads to sample to eliminate shorter fragments and concentrate the DNA. The concentration of the sheared and purified DNA was assessed using a Nanodrop spectrophotometer and Qubit Fluorometer and Qubit dsDNA High Sensitivity Assay kit. Fragment size distribution was evaluated by running the sample on the FemtoPulse system.

RNA was extracted from abdomen tissue of iyMegLign3 in the Tree of Life Laboratory at the WSI using the RNA Extraction: Automated MagMax™ *mir*Vana protocol ( do Amaral *et al.*, 2023). The RNA concentration was assessed using a Nanodrop spectrophotometer and a Qubit Fluorometer using the Qubit RNA Broad-Range Assay kit. Analysis of the integrity of the RNA was done using the Agilent RNA 6000 Pico Kit and Eukaryotic Total RNA assay.

### Sequencing

Pacific Biosciences HiFi circular consensus DNA sequencing libraries were constructed according to the manufacturers’ instructions. Poly(A) RNA-Seq libraries were constructed using the NEB Ultra II RNA Library Prep kit. DNA and RNA sequencing was performed by the Scientific Operations core at the WSI on Pacific Biosciences SEQUEL II (HiFi) and Illumina NovaSeq 6000 (RNA-Seq) instruments. Hi-C data were also generated from head and thorax tissue of iyMegLign2 using the Arima2 kit and sequenced on the Illumina NovaSeq 6000 instrument.

### Genome assembly, curation and evaluation

Assembly was carried out with Hifiasm ( Cheng *et al.*, 2021) and haplotypic duplication was identified and removed with purge_dups ( Guan *et al.*, 2020). The assembly was then scaffolded with Hi-C data ( Rao *et al.*, 2014) using YaHS ( Zhou *et al.*, 2023). The assembly was checked for contamination and corrected using the gEVAL system ( Chow *et al.*, 2016) as described previously ( Howe *et al.*, 2021). Manual curation was performed using gEVAL, HiGlass ( Kerpedjiev *et al.*, 2018) and Pretext ( Harry, 2022). The mitochondrial genome was assembled using MitoHiFi ( Uliano-Silva *et al.*, 2023), which runs MitoFinder ( Allio *et al.*, 2020) or MITOS ( Bernt *et al.*, 2013) and uses these annotations to select the final mitochondrial contig and to ensure the general quality of the sequence.

A Hi-C map for the final assembly was produced using bwa-mem2 ( Vasimuddin *et al.*, 2019) in the Cooler file format ( Abdennur & Mirny, 2020). To assess the assembly metrics, the *k*-mer completeness and QV consensus quality values were calculated in Merqury ( Rhie *et al.*, 2020). This work was done using Nextflow ( Di Tommaso *et al.*, 2017) DSL2 pipelines “sanger-tol/readmapping” ( Surana *et al.*, 2023a) and “sanger-tol/genomenote” ( Surana *et al.*, 2023b). The genome was analysed within the BlobToolKit environment ( Challis *et al.*, 2020) and BUSCO scores ( Manni *et al.*, 2021; Simão *et al.*, 2015) were calculated.

Table 3 contains a list of relevant software tool versions and sources.

**Table 3.** Software tools: versions and sources.

Software tool	Version	Source
BlobToolKit	4.1.7	https://github.com/blobtoolkit/blobtoolkit
BUSCO	5.3.2	https://gitlab.com/ezlab/busco
gEVAL	N/A	https://geval.org.uk/
Hifiasm	0.16.1-r375	https://github.com/chhylp123/hifiasm
HiGlass	1.11.6	https://github.com/higlass/higlass
Merqury	MerquryFK	https://github.com/thegenemyers/MERQURY.FK
MitoHiFi	2	https://github.com/marcelauliano/MitoHiFi
PretextView	0.2	https://github.com/wtsi-hpag/PretextView
purge_dups	1.2.3	https://github.com/dfguan/purge_dups
sanger-tol/genomenote	v1.0	https://github.com/sanger-tol/genomenote
sanger-tol/readmapping	1.1.0	https://github.com/sanger-tol/readmapping/tree/1.1.0
YaHS	yahs-1.1.91eebc2	https://github.com/c-zhou/yahs

### Genome annotation

The Ensembl Genebuild system ( Aken *et al.*, 2016) was used to generate annotation for the *Megachile ligniseca* assembly (GCA_945859555.1) at the EBI. Annotation was created primarily through alignment of transcriptomic data to the genome, with gap filling via protein-to-genome alignments of a select set of proteins from UniProt ( UniProt Consortium, 2019).

### Wellcome Sanger Institute – Legal and Governance

The materials that have contributed to this genome note have been supplied by a Darwin Tree of Life Partner. The submission of materials by a Darwin Tree of Life Partner is subject to the **‘Darwin Tree of Life Project Sampling Code of Practice’**, which can be found in full on the Darwin Tree of Life website here. By agreeing with and signing up to the Sampling Code of Practice, the Darwin Tree of Life Partner agrees they will meet the legal and ethical requirements and standards set out within this document in respect of all samples acquired for, and supplied to, the Darwin Tree of Life Project. 

Further, the Wellcome Sanger Institute employs a process whereby due diligence is carried out proportionate to the nature of the materials themselves, and the circumstances under which they have been/are to be collected and provided for use. The purpose of this is to address and mitigate any potential legal and/or ethical implications of receipt and use of the materials as part of the research project, and to ensure that in doing so we align with best practice wherever possible. The overarching areas of consideration are:

• Ethical review of provenance and sourcing of the material

• Legality of collection, transfer and use (national and international) 

Each transfer of samples is further undertaken according to a Research Collaboration Agreement or Material Transfer Agreement entered into by the Darwin Tree of Life Partner, Genome Research Limited (operating as the Wellcome Sanger Institute), and in some circumstances other Darwin Tree of Life collaborators.

## Data Availability

European Nucleotide Archive: *Megachile ligniseca* (wood-carving leafcutter bee). Accession number PRJEB53608; https://identifiers.org/ena.embl/PRJEB53608 ( Wellcome Sanger Institute, 2022). The genome sequence is released openly for reuse. The *Megachile ligniseca* genome sequencing initiative is part of the Darwin Tree of Life (DToL) project. All raw sequence data and the assembly have been deposited in INSDC databases. Raw data and assembly accession identifiers are reported in Table 1.

## Author information

Members of the University of Oxford and Wytham Woods Genome Acquisition Lab are listed here: https://doi.org/10.5281/zenodo.7125292.

Members of the Darwin Tree of Life Barcoding collective are listed here: https://doi.org/10.5281/zenodo.4893703.

Members of the Wellcome Sanger Institute Tree of Life Management, Samples and Laboratory team are listed here: https://doi.org/10.5281/zenodo.10066175.

Members of Wellcome Sanger Institute Scientific Operations: Sequencing Operations are listed here: https://doi.org/10.5281/zenodo.10043364.

Members of the Wellcome Sanger Institute Tree of Life Core Informatics team are listed here: https://doi.org/10.5281/zenodo.10066637.

Members of the Tree of Life Core Informatics collective are listed here: https://doi.org/10.5281/zenodo.5013541.

Members of the Darwin Tree of Life Consortium are listed here: https://doi.org/10.5281/zenodo.4783558.
