# Supplementary material for: The genome sequence of the wood-carving leafcutter bee, Megachile ligniseca (Kirby, 1802)
Source: Wellcome Open Res. 2024 Mar 1;9:103. [Version 1] doi: 10.12688/wellcomeopenres.21002.1 (PMC11187525; doi:10.12688/wellcomeopenres.21002.1)
Supplement: Peer review history

# Reviewer report: Reviewer response for version 1

**DOI:** 10.21956/wellcomeopenres.23237.r85127

**Published:** 2024-06-19

**Authors:** Zheng Li, Allen Xue

**Affiliations:** 1 The University of Texas at Austin, Austin, Texas, USA

In this manuscript, the authors provide a chromosome-level assembly for the wood-carving leafcutter bee, *Megachile ligniseca*. The genome report is clearly described, with the necessary genome statistics and a table of the chromosome-level scaffolds given, allowing the reader to understand the basic size and quality of the genome. The methods for identification of the specimen and extraction and processing of HMW DNA were also concisely outlined and easy to follow. Software tools used for genome analysis are also provided in an organized manner.

 Minor points:

 The background information has a few typos (“In the UK is has” should be “In the UK it has”; “Females have a buff-coloured scopal hairs” should be “Females have buff-coloured scopal hairs”) and in general should be more clearly written to make it easier for a reader not familiar with bee biology to understand the characteristics of *M. ligniseca.*

 The methods section could use a little more detail in describing the protocol. It may be easier to follow if some of the more crucial steps are explicitly stated in this section.

Are sufficient details of methods and materials provided to allow replication by others?

Yes

Is the rationale for creating the dataset(s) clearly described?

Yes

Are the datasets clearly presented in a useable and accessible format?

Yes

Are the protocols appropriate and is the work technically sound?

Partly

Reviewer Expertise:

Genomics, Evolutionary Biology

We confirm that we have read this submission and believe that we have an appropriate level of expertise to confirm that it is of an acceptable scientific standard.

# Reviewer report: Reviewer response for version 1

**DOI:** 10.21956/wellcomeopenres.23237.r83606

**Published:** 2024-06-12

**Authors:** Arong Luo

**Affiliations:** 1 University of the Chinese Academy of Sciences, Beijing, Beijing, China

The *Megachilidae* family is the second largest under *Apoidea*, distinguished by the unique pollen brush on the underside of their abdomen, which is a specialized structure for pollination. These bees are crucial pollinators for leguminous forage and fruit trees, holding significant economic value. As solitary bees, they possess strong pollination abilities and serve as excellent models for studying solitary pollinators. The *Megachile rotundata*, in particular, is a highly efficient pollinator of Medicago sativa and the most important insect pollinator for alfalfa. Consequently, leaf-cutting bees are vital for studying the coevolution of insects and plants. Deciphering the genome of *Megachilidae* will provide a valuable genomic resource for future research on the evolution and ecology of wild pollinators.

*Megachile ligniseca *is a leaf-cutting bee primarily found in Europe. In this study, a chromosome-scale genome of *Megachile ligniseca* was assembled using high-throughput sequencing technology. The genome size of this species was found to be 290Mb, with a total of 16 chromosomes, consistent with most *Apis* and *Megachile* bees. The study's methodology is clearly described, complemented by detailed charts, making it a classic example of genome research.

 There are three suggestions and questions:

 1) The species photos of *Megachile ligniseca* in the article should include morphological features.

 2) In *Apis*, male bees are haploid, and many bee genomes use males as sequencing material. Why did you not choose females for sequencing?

 3) The genome size assembled by Hifiasm is 100Mb larger than expected. Why did you insist on using Hifiasm? Although using purge_dups to eliminate redundancy improved the results, was the purging process excessive?

Are sufficient details of methods and materials provided to allow replication by others?

Yes

Is the rationale for creating the dataset(s) clearly described?

Yes

Are the datasets clearly presented in a useable and accessible format?

Yes

Are the protocols appropriate and is the work technically sound?

Yes

Reviewer Expertise:

Molecular phylogenetics and evolution

I confirm that I have read this submission and believe that I have an appropriate level of expertise to confirm that it is of an acceptable scientific standard.
